# A novel hybrid method with convergence analysis for approximation of HTLV-I dynamics model

**DOI:** 10.1038/s41598-024-76110-9

**Published:** 2024-10-27

**Authors:** Mahboubeh Molavi-Arabshahi, Jalil Rashidinia, Mahnaz Yousefi

**Affiliations:** School of Mathematics and Computer Science, Iran University of Science and Technology, Narmak, Tehran 16844 Iran

**Keywords:** Legendre polynomials, HTLV-I, Spectral collocation method, Operational matrix, The nonlinear system, Biophysics, Computational biology and bioinformatics, Diseases, Mathematics and computing

## Abstract

This paper presents a novel numerical approach for approximating the solution of the model describing the infection of $CD4^+T$-cells by the human T-cell lymphotropic virus I (HTLV-I).The proposed method utilizes the operational matrix along with spectral method to convert the fractional model into a system of nonlinear algebraic equations. The Levenberg-Marquardt algorithm efficiently solves these equations. The study includes theoretical convergence analysis and error bounds to establish the validity of the proposed method. Through several test problems, we demonstrate the effectiveness and accuracy of the approach. We compare its performance and reliability to other existing methods in the literature. The results indicate that the proposed method is a reliable and efficient approach for solving the model.

## Introduction

**Fig. 1:**
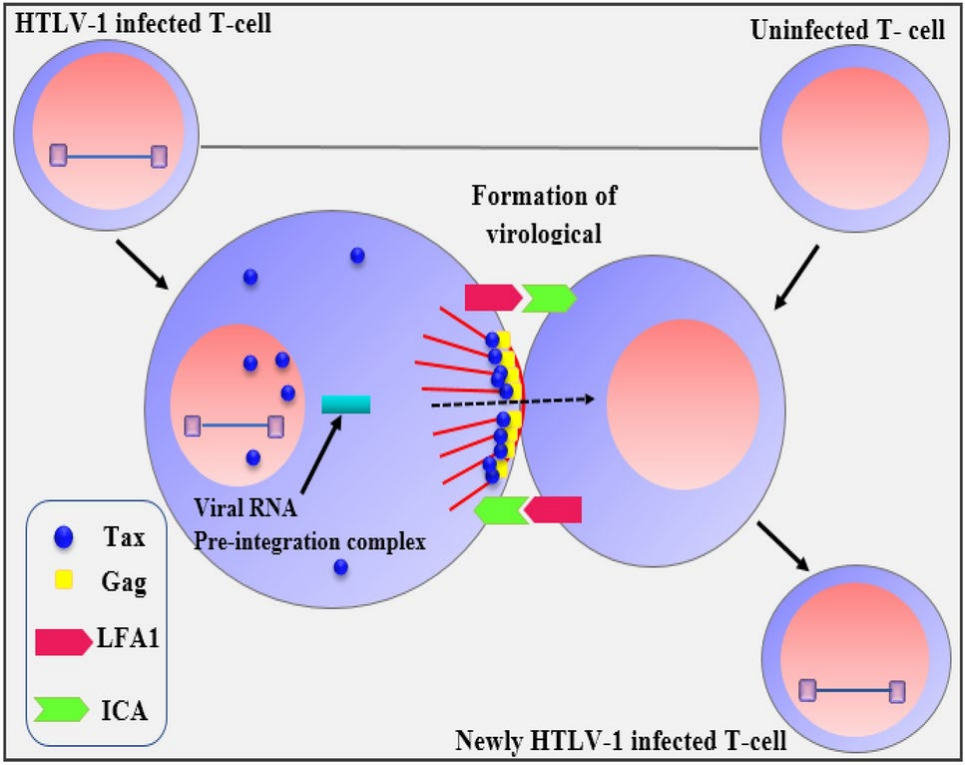
HTLV-I transmission via cell contact.

According to statistics from the World Health Organization (WHO), approximately 20 million individuals worldwide are affected by HTLV-I infection^1^. The disease progresses slowly within the human body and is considered an incurable lifelong condition^2,3^. It can be transmitted through various bodily fluids, including breast milk, semen, and blood^4^. There are three main routes of transmission: mother-to-infant transmission,^5^ parenteral transmission^6,7^, and sexual contact^8^. It is a type of virus that infects humans (retrovirus) and carries its genetic information in a single-stranded RNA molecule (ribonucleic acid), specifically infects $CD4^+T$-cells and propagates through cell-to-cell transmission from infected cells to naive cells^9,10^. Because HTLV-I spreads through direct contact between cells, infected cells need to be passed on from an infected person for transmission to occur, through one of the specific routes. When an infected cell interacts with an uninfected cell, a microtubule organizing center (MTOC) becomes polarized, leading to the formation of a virological synapse. During HTLV-I infection, a special junction forms between an infected cell and a healthy cell, called a synapse^11^. Following this step, a latency period ensues, during which infected cells harbor the virus without actively producing viral DNA. Consequently, these cells are unable to transmit the infection. However, upon stimulation by antigens, latently infected cells can become activated and infect healthy cells (see Fig. 1)^12^.

Numerous models have been proposed to capture the behavior of the human immune system in the presence of HTLV-I^13–18^. Mathematical modeling provides a powerful tool for investigating the complex dynamics of HTLV-1 infection and developing strategies to combat this disease. By combining experimental data with theoretical models, researchers can gain valuable insights into the biology of HTLV-I and inform the development of effective interventions.^19,20^. A mathematical model proposed by Stilianakis^21^ categorizes $CD4^+T$-cells into four distinct populations using a system of nonlinear differential equations. Katri et al.^22^ introduced modifications to the model^21^ as follows:

1 $$\begin{aligned} \left\{ \begin{array}{lc} H^{\prime }(t)=\mu -\eta _HH(t)-kV(t)H(t),\quad & H(0)=\nu _1,\\ I^{\prime }(t)= k_1 V(t)H(t)-(\gamma +\eta _L)I(t),\quad & I(0)=\nu _2,\\ V^{\prime }(t)= \gamma I(t)-(\kappa +\eta _A)V(t),\quad & V(0)=\nu _3,\\ L^{\prime }(t)=\kappa V(t)+sL(t)(1-\frac{L(t)}{(L)_{max}})-\eta _NL(t)\quad & L(0)=\nu _4. \end{array}\right. \end{aligned}$$

Where *H*(*t*), *I*(*t*), *V*(*t*), and *L*(*t*) represent the number of healthy cells, latently infected cells, actively infected cells, and leukemic cells at time *t*, respectively. The model shows how HTLV-I infection develops over time. A detailed description of the factors involved in the HTLV-I model is provided in Table 1.

**Table 1:** HTLV-I model variables and parameters.

Parameters	Description
$\eta _N$	Mortality rate of leukemic infected $CD4^+T$-cells
$\eta _A$	Mortality rate of actively infected $CD4^+T$-cells
$\eta _L$	Mortality rate of latently infected $CD4^+T$-cells
$\eta _H$	Mortality rate of healthy $CD4^+T$-cells
$k_1$	The rate at which virus is transmitted to $CD4^+T$-cells from actively infected cells
*k*	The rate at which actively infected cells come into contact with uninfected cells
$\mu$	The origin of $CD4^+T$-cells
*s*	The growth rate of ATL cells
$\kappa$	The rate at which actively infected cells transition into leukemic cells
$\gamma$	The rate at which latently infected cells transition into actively leukemic cells
$\tau$	A positive constant
$(L)_{max}$	Maximum number of leukemic $CD4^+T$-cells

Fractional models incorporate memory into their calculations, meaning that the system’s state is influenced not only by present conditions but also by its history. The strength of this memory effect is represented by a fractional order, where a higher order indicates a slower decay of past influences on the system’s behavior. This memory aspect allows fractional models to reflect real-world phenomena with inherent historical dependencies. Numerical methods are essential for approximating solutions to fractional differential equations^23–25^ and integro-differential equations^26,27^, as analytical solutions are often difficult or impossible to obtain. Current techniques include finite difference methods^28^, finite element methods^29^, spectral methods^30^, and fractional calculus-based methods^31^. These methods involve discretizing the fractional derivatives or integrals and solving the resulting system of equations using numerical techniques. The choice of method depends on the specific problem, the desired accuracy, and computational efficiency considerations. Due to the lack of exact solutions for models of real-world phenomena, many mathematicians have focused on developing accurate approximations^32,33^.

Motivated by the aforementioned research, this paper introduces a numerical method for solving the Caputo fractional HTLV-I model.

2 $$\begin{aligned} \left\{ \begin{array}{l} D^\zeta H(t)=\mu -\eta _H H(t)-k V(t) H(t), \\ D^\zeta I(t)=k_1 V(t) H(t)-\left( \gamma +\eta _L\right) I(t), \\ D^\zeta V(t)=\gamma I(t)-\left( \kappa +\eta _A\right) V(t), \\ D^\zeta L(t)=\kappa V(t)+s L(t)\left( 1-\frac{L(t)}{\left( L\right) _{\max }}\right) -\eta _N L(t), \end{array}\right. \end{aligned}$$

with initial conditions

3 $$\begin{aligned} H(0)=\nu _1, I(0)=\nu _2,V(0)=\nu _3,L(0)=\nu _4. \end{aligned}$$

Several numerical methods have been applied to estimate the solution of the HTLV-I model, like the natural-Adomian decomposition method (N-ADM)^34^, the multi-step generalized differential transform method^35^, and the generalized Euler method^36^. Although effective, these methods have limitations regarding computational complexity and accuracy. To overcome these challenges, we propose an alternative approach that utilizes the Legendre operational matrix and spectral methods, transforming the fractional HTLV-I model into a system of nonlinear algebraic equations. We employ the Levenberg-Marquardt algorithm to solve this system, ensuring efficiency and accuracy. The study includes theoretical convergence analysis and error bounds to validate the proposed method.

Previous studies have demonstrated the effectiveness of the Legendre operational matrix in solving a range of fractional differential equations, including fractional anomalous diffusion equations^37^, fractional partial differential equations with variable coefficients^38^, and distributed-order fractional differential equations^39^. Building on this foundation, we extend the use of the Legendre operational matrix in conjunction with spectral methods to solve the fractional HTLV-I model. The proposed method offers two key advantages. Firstly, the derivative operational matrix based on the shifted Legendre polynomials is sparse, reducing computational costs. Secondly, using sparse Legendre operational matrices for derivatives alongside the collocation spectral method results in high accuracy and fast computation times. The rest of this article is structured as follows:

In Sect. “Preliminaries”, some essential concepts in fractional calculus and the properties of shifted Legendre polynomials (SLPs) are given. In Sect. “The proposed method”, the numerical algorithm to estimate the solution to the model is presented. In Sect. “Error analysis”, the convergence analysis of the numerical method is discussed. In Sect. “Numerical results”, the proposed method is evaluated using several numerical examples. The results are then compared with previously reported methods in the existing literature. Finally, a brief remark is presented.

## Preliminaries

This section provides a brief overview of the essential principles underlying fractional calculus and Legendre polynomials^40^.

### Definition 1

The Riemann-Liouville fractional integral of order $\zeta >0$ is defined as follows:

4 $$\begin{aligned} \begin{aligned}&I^0 \hat{f}(t)=\hat{f}(t), \\&I^\zeta \hat{f}(t)=\frac{1}{\Gamma (\zeta )} \int _0^{t}\hat{f}(s)(t-s)^{(\zeta -1)} ds=\frac{1}{\Gamma (\zeta )} t^{\zeta -1} * \hat{f}(t), \quad t>0,\zeta >0, \end{aligned} \end{aligned}$$

where the convolution operator and the gamma function are represented by $*$ and $\Gamma (.)$, respectively

### Definition 2

The following formulas determine the Caputo fractional derivative of order $\zeta$ as:

5 $$\begin{aligned} \begin{aligned} D^\zeta \hat{f}(t)=I^{n-\zeta } D^{n} \hat{f}(t)&=\frac{1}{\Gamma (n-\zeta )} \int _0^{t}(t-s)^{n-\zeta -1} \hat{f}^{(n)}(s)ds, \\ n-1&<\zeta \le n, t>0, n \in \mathbb {N}. \end{aligned} \end{aligned}$$

specially, we have

6 $$\begin{aligned} D_{t}^\zeta t^n= {\left\{ \begin{array}{ll}\frac{\Gamma (n+1)}{\Gamma (n+\zeta +1)} t^{n-\zeta }, &  n \ge \zeta \\ 0, &  n<\zeta \end{array}\right. } \end{aligned}$$

### Definition 3

The following space

7 $$\begin{aligned} B^{n} (\Omega )=\{f: \frac{\partial ^{k}f}{\partial t^{k}} \in L^2(\Omega )\}, \end{aligned}$$

with

$$(f,u)_{B^{n}}=\sum _{k=0}^{n}\left( \frac{\partial ^{k}f}{\partial t^{k}}, \frac{\partial ^{k}u}{\partial t^{k}} \right) , \Vert f \Vert _{B^{n}}=(f,f)_{B^{n}}^{\frac{1}{2}}.$$

is called the Legendre weighted Sobolev space.

where $k= 0,1,\dots ,n$, $\Omega =[0,\tau ]$. Note that $H^n(\Omega )$ is a subspace of (7), i.e.,

$$\Vert f \Vert _{B^{n}} \le c \Vert f \Vert _{H^{n}}, n\ge 0.$$

### The legendre polynomial

The following is the recurrence relation used to determine the classical Legendre polynomials for $t \in [-1,1]$:

8 $$\begin{aligned} \begin{aligned}&\hat{L}_0(t)=1, \quad \hat{L}_1(t)=t, \\&\hat{L}_{s+1}(t)=\frac{2 s+1}{s+1} t \hat{L}_{s}(t)-\frac{s}{s+1} \hat{L}_{s-1}(t). \quad s=1,2, \ldots \,\,, \end{aligned} \end{aligned}$$

The shifted Legendre polynomial (SLP) is expressed by the substituting $z = \frac{2t - \tau }{\tau }$ in Eq. (8) as follows:

9 $$\begin{aligned} \hat{L}_{\tau ,s}(t)=\hat{L}_{s}\left( \frac{2t}{\tau }-1\right) , \quad 0 \le t \le \tau . \end{aligned}$$

where $$s=0,1, \ldots \,\,$$.

Thus, we have:

10 $$\begin{aligned} \begin{aligned}&\hat{L}_{\tau ,0}(t)=1, \quad \hat{L}_{\tau , 1}(t)=\frac{2t}{\tau }-1\\&\hat{L}_{\tau ,s+1}(t)=\frac{(2t-\tau )(2s+1)}{\tau (s+1)} \hat{L}_{\tau ,s}(t)-\frac{s}{1+s} \hat{L}_{\tau , s-1}(t), \quad s=1,2, \ldots \,. \end{aligned} \end{aligned}$$

The analytical form of the above expression is obtained as follows:

11 $$\begin{aligned} \hat{L}_{\tau ,s}(t)=\sum _{c=0}^{s}(-1)^{s+c} \frac{(s+c)!}{(s-c)!(c!)^2 \tau ^{c}} t^{c}. \end{aligned}$$

It is worth mentioning that the SLPs form a complete orthogonal system, i.e.,

12 $$\begin{aligned} \int _0^\tau \hat{L}_{\tau ,s}(t) \hat{L}_{\tau ,i}(t) dt=\frac{\tau }{2s+1} \delta _{si},\, s,i=0,1,..., \end{aligned}$$

where $\delta _{si},$ denotes the Kronecker delta.

### Approximation function

Any smooth function *f*(*t*) defined on the interval $[0,\tau ]$ can be represented as:

13 $$\begin{aligned} f(t)=\sum _{s=0}^{\infty } b_{s} \hat{L}_{\tau ,s}(t), \end{aligned}$$

where

14 $$\begin{aligned} b_{s}=\left( \frac{2s+1}{\tau }\right) \int _0^\tau f(t) \hat{L}_{\tau ,s}(t)dt, \end{aligned}$$

In the application, we only take into account the initial (N+1)-terms of the SLPs, i.e.:

15 $$\begin{aligned} f(t)=\sum _{s=0}^{N} b_{s} \hat{L}_{\tau ,s}(t)=B\psi _{\tau , N}(t), \end{aligned}$$

where $\psi _{\tau , N}(t)=\left[ \hat{L}_{\tau , 0}(t), \hat{L}_{\tau ,1}(t), \ldots , \hat{L}_{\tau ,N}(t)\right] ^T$ and $B=\left[ b_0, b_1,b_2, \ldots ,b_N\right]$. The operational matrix formulated based on the SLPs is structured as follows:

16 $$\begin{aligned} \psi _{\tau ,N}(t)=\textbf{P}\mathbf {T_N(t)}, \end{aligned}$$

where

17 $$\begin{aligned} \psi _{\tau , N}(t)=\left[ \hat{L}_{\tau , 0}(t), \hat{L}_{\tau ,1}(t), \ldots , \hat{L}_{\tau ,N}(t)\right] ^T,\quad \mathbf {T_N(t)}=\left[ 1, t, t^2, \ldots , t^N\right] ^T, \end{aligned}$$

and

18 $$\begin{aligned} \textbf{P}=\left( \begin{array}{ccccc} 1 & 0 & 0 & \cdots & 0 \\ -1 & (-1)^22! & 0 & \cdots & 0 \\ (-1)^2 & (-1)^3 \frac{3!}{1!} & (-1)^4\frac{4!}{2!} & \cdots & 0 \\ \vdots & \vdots & \vdots & \ddots & \vdots \\ (-1)^N & (-1)^{N+1} \frac{(N+1)!}{(N-1)!} & (-1)^{N+2} \frac{(N+2)!}{2!(N-2)!} & \cdots & (-1)^{2N} \frac{(2N)!}{(N)!} \end{array}\right) . \end{aligned}$$

Since $\textbf{P}$ is non-singular, we have

19 $$\begin{aligned} \mathbf {T_N(t)}=\textbf{P}^{-1}\psi _{\tau ,N}(t). \end{aligned}$$

Next, we calculate the derivative of $\psi _{\tau ,N}(t)$ as follows:

20 $$\begin{aligned} \frac{d\psi _{\tau ,N}(t)}{dt}=\mathbf {D^{\textbf {1}}}\psi _{\tau ,N}(t), \end{aligned}$$

where

21 $$\begin{aligned} \mathbf {D^1}=[d_{jk}^1]_{(N+1)\times (N+1)},\quad d_{jk}^1= {\left\{ \begin{array}{ll}k, & k=j-1,j=2,...,N+1, \\ 0, & otherwise,\end{array}\right. }\quad j,k=1,...,N+1. \end{aligned}$$

It is clear that, employing Eq. (16), we obtain

22 $$\begin{aligned} \frac{d^n\psi _{\tau ,N}(t)}{dt^n}=\mathbf {D^{\textbf {(n)}}}\psi _{\tau ,N}(t), \end{aligned}$$

where $$\mathbf {D^{\textbf {(n)}}}=(\mathbf {D^{\textbf {(1)}}})^{(n)}$$ and $$n\in \mathbb {N}$$ .

## The proposed method

Here, we develop a spectral collocation technique along with the operational matrix that relies on SLPs in the temporal dimension to solve the problem (2)–(3) in the following steps.

- *Step* 1. We employ SLPs as an approximation technique, as referred to in Eq. (15), to estimate the solutions of system (2) in matrix forms:
  23 $$\begin{aligned} {\left\{ \begin{array}{ll} & H(t) \simeq \sum _{s=0}^N b_{1,s} \hat{L}_{\tau ,s}(t)=B_{1}\psi _{\tau ,N}(t),\\ & I(t) \simeq \sum _{s=0}^N b_{2,s} \hat{L}_{\tau ,s}(t)=B_{2}\psi _{\tau ,N}(t),\\ & V(t) \simeq \sum _{s=0}^N b_{3,s} \hat{L}_{\tau ,s}(t)=B_{3}\psi _{\tau ,N}(t),\\ & L(t) \simeq \sum _{s=0}^N b_{4,s} \hat{L}_{\tau ,s}(t)=B_{4}\psi _{\tau ,N}(t),\\ \end{array}\right. } \end{aligned}$$
  where $B_{1}=[b_{1,0},b_{1,1},b_{1,3},...,b_{1,N}]$, $B_{2}=[b_{2,0},b_{2,1},b_{2,3},...,b_{2,N}]$, $B_{3}=[b_{3,0},b_{3,1},b_{3,3},...,b_{3,N}]$ and $B_{4}=[b_{4,0},b_{4,1},b_{4,3},...,b_{4,N}]$ are unknown vectors.
  By substituting Eqs. (16) in Eq. (23), we obtain
  24 $$\begin{aligned} {\left\{ \begin{array}{ll} & H(t) \simeq B_{1} \textbf{P}\mathbf {T_N(t)},\\ & I(t) \simeq B_{2}\textbf{P} \mathbf {T_N(t)}, \\ & V(t) \simeq B_{3}\textbf{P} \mathbf {T_N(t)}, \\ & L(t) \simeq B_{4}\textbf{P} \mathbf {T_N(t)}, \\ \end{array}\right. } \end{aligned}$$
  Therefore,
  25 $$\begin{aligned} \mathbf {U(t)}=\left[ \begin{array}{c} H(t) \\ I(t) \\ V(t) \\ L(t) \end{array}\right] =\mathbf {BPT_N(t)}, \end{aligned}$$
  where
  26 $$\begin{aligned} \textbf{B}=[B_1,B_2,B_3,B_4]. \end{aligned}$$
- *Step* 2. By employing Eqs. (6) and (24), we have:
  27 $$\begin{aligned} \begin{aligned} \frac{d^\zeta (H(t))}{d t^\zeta }&\simeq B_{1} \textbf{P} \frac{d^\zeta }{d t^\zeta }\left( \mathbf {T_N(t)}\right), \\&=B_{1}\textbf{P}\mathbf {\mathbf {W_{t}^{\zeta }}}\mathbf {T_N(t)}, \end{aligned} \end{aligned}$$
  where
  28 $$\begin{aligned} \begin{aligned} \mathbf {\mathbf {W_{t}^{\zeta }}}=[w_{jk}^{\zeta }]_{(N+1)\times (N+1)},\\w_{jk}^{\zeta }&= {\left\{ \begin{array}{ll}\frac{\Gamma (j)}{\Gamma (j-\zeta )}t^{-\zeta }, & j=k,j=2,...,N+1, \\ 0, & otherwise,\end{array}\right. }\, j,k=1,...,N+1. \end{aligned} \end{aligned}$$
  Using a similar procedure for *I*(*t*), *V*(*t*) and *L*(*t*) the following results hold as
  29 $$\begin{aligned} {\left\{ \begin{array}{ll} & \frac{d^\zeta (H(t))}{d t^\zeta }\simeq B_{1} \textbf{P}\mathbf {\mathbf {W_{t}^{\zeta }}T_{N}(t)},\\ & \frac{d^\zeta (I(t))}{d t^\zeta }\simeq B_{2}\textbf{P}\mathbf {\mathbf {W_{t}^{\zeta }}T_{N}(t)},\\ & \frac{d^\zeta (V(t))}{d t^\zeta }\simeq B_{3}\textbf{P}\mathbf {\mathbf {W_{t}^{\zeta }}T_{N}(t)},\\ & \frac{d^\zeta (L(t))}{d t^\zeta }\simeq B_{4}\textbf{P}\mathbf {\mathbf {W_{t}^{\zeta }}T_{N}(t)},\\ \end{array}\right. } \end{aligned}$$
  Therefore,
  30 $$\begin{aligned} \mathbf {U^{(\zeta )}(t)}=\left[ \begin{array}{c} H^{(\zeta )}(t) \\ I^{(\zeta )}(t)\\ V^{(\zeta )}(t)\\ L^{(\zeta )}(t) \end{array}\right] =\mathbf {BPW_{t}^{\zeta }T_N(t).} \end{aligned}$$
- *Step* 3. According to the results obtained from steps 1 and 2, we can express the model (2) in the matrix form as follows:
  31 $$\begin{aligned} \mathbf {U^{(\zeta )}(t)}=\mathbf {QU(t)+KC(t)+M\bar{U}(t)U(t)+Y}, \end{aligned}$$
  where
  $$\begin{aligned} \mathbf {U^{(\zeta )}(t)}=\left[ \begin{array}{c} H^{(\zeta )}(t)\\ I^{(\zeta )}(t)\\ V^{(\zeta )}(t)\\ L^{(\zeta )}(t) \end{array}\right] ,\mathbf {U(t)}=\left[ \begin{array}{c} H(t)\\ I(t)\\ V(t)\\ L(t) \end{array}\right] \text{, }\bar{\textbf{U}}= \left( \begin{array}{cccc} H(t) & 0 & 0 & 0 \\ 0 & I(t) & 0 & 0 \\ 0& 0 & V(t) & 0 \\ 0 & 0 & 0 & L(t) \end{array}\right) \text{, } \end{aligned}$$
  $$\begin{aligned} \textbf{Q}= \left( \begin{array}{cccc} -\eta _H & 0 & 0 & 0 \\ 0 & -(\eta _L+\gamma ) & 0 & 0 \\ 0& +\gamma & -\eta _A+\kappa & 0 \\ 0 & 0 & 0 & \kappa -\eta _N \end{array}\right) \text{, }\textbf{M}= \left( \begin{array}{cccc} \frac{-s}{L_{max}} & 0 & 0 & 0 \\ 0 & 0 & 0 & 0 \\ 0& 0 & 0& 0 \\ 0 & 0 & 0 & 0 \end{array}\right) \text{, }\textbf{K}=\left[ \begin{array}{c} -k \\ k_1 \\ 0 \\ 0 \end{array}\right] \text{, } \end{aligned}$$
  $$\begin{aligned} \textbf{Y}=\left[ \begin{array}{c} \mu \\ 0 \\ 0 \\ 0 \end{array}\right] \text{ and }\,\mathbf {C(t)}=\left[ V(t)T(t)\right] \text{. } \end{aligned}$$
- *Step* 4. In order to ascertain the values of the unknown coefficients $b_{1,n}$, $b_{2,n}$, $b_{3,n}$, and $b_{4,n}$ (where *n* ranges from 0 to *N*), we consider the roots of $\hat{L}_{\tau ,N-1}(t)$ as collocation nodes, represented by $t_i,\,i=0,1,\dots ,N-1$. By substituting the collocation points into Eq. (31), we derive a system of $4\times (N)$ matrix equations:
  32 $$\begin{aligned} \mathbf {U^{(\zeta )}(t_i)}=\mathbf {QU(t_i)+KC(t_i)+M\bar{U}(t_i)U(t_i)+Y},\quad i=0,1,...,N \end{aligned}$$
  To solve system (32) uniquely, four additional equations are required. Thus, we replace Eq. (24) in the initial conditions (3) and assume $t_0=0$ as follows:
  33 $$\begin{aligned} \mathbf {U(0)}=\mathbf {\nu } .\end{aligned}$$
  where $\mathbf {\nu }=[\nu _1,\nu _2,\nu _3,\nu _4].$ Eq. (32) along with Eq. (33) form a system of $4\times (N+1)$ nonlinear algebraic equations which is solved by the Levenberg-Marquardt algorithm. By solving this system, the unknown coefficients $b_{1,n}$, $b_{2,n}$, $b_{3,n}$, and $b_{4,n}$ are determined.
- *Step* 5. In the end, by substituting vectors $B_1$, $B_2$, $B_3$ and $B_4$ in Eq. (23), we estimate the solution of HTLV-I model (2)–(3).

## Error analysis

In this section, we compute an error bound for the approximate solution obtained in Sect. “The proposed method”. Let $\Pi _{N}f$ be the operator that maps $L^2(\Omega )$ into $P_N(\Omega )$, defined as follows:

$$(\Pi _{N}f-f,v)=0, \forall v\in P_N(\Omega ),\Omega =[0,\tau ],$$

where

$$P_N(\Omega )=span\{\hat{L}_{\tau ,0},\hat{L}_{\tau ,1},\dots ,\hat{L}_{\tau ,N}\},\, N\in \mathbb {N}.$$

in other words,

$$(\Pi _Nf)(t)=\sum _{i=0}^{N}a_i\hat{L}_{\tau ,i}(t),$$

$\Pi _{N}f$ is the best approximation of *f* out of $\Pi _{N}f$ as mentioned in (^41^). First, the following theorems need to be proven.

### Theorem 1

*Suppose*$\Omega =[0,\tau ]$* and*$f \in H^n(\Omega )$* as defined in Definition*3.* There exists a constant*$\hat{C}_1$* such that:*

34 $$\begin{aligned} \left\| \Pi _N f-f\right\| _{L^2(\Omega )} \le \hat{C}_1 N^{-n}\left\| f\right\| _{H^n(\Omega )}, \end{aligned}$$

### Proof

^42^ $$\square$$

### Theorem 2

*For any*$f\in B^n (\Omega )$* as introduced in Definition*3,* we have*

35 $$\begin{aligned} \begin{aligned} \left\| D_{t}^{n}\left( \Pi _{N} f-f\right) \right\|&\le \hat{C_2} N^{(p-n)}\left\| D_{t}^{n} f\right\| , \\&\le \hat{C_2} N^{(p-n)}\Vert f\Vert, \end{aligned} \end{aligned}$$

*where*$\hat{C_2}$* is constant and*$0 \le p\le n \le N+1,\, n\in \mathbb {N}.$

*In Hilbert space, the following relationship holds:*

36 $$\begin{aligned} \left\| D_{t}^{n}\left( \Pi _{N} f-f\right) \right\| \le \hat{C_2}N^{(p-n)}\Vert f\Vert _{H^{n}}. \end{aligned}$$

### Proof

^41^ $$\square$$

Now, we compute an error bound in the estimation of $D_{t}^\zeta f$ as follows.

### Theorem 3

*Let*$f\in L^2(\Omega ),p<r\le N+1$, $p-1<\zeta \le p=\lceil \zeta \rceil$, *and*$r\in \mathbb {N}$*then,*

37 $$\begin{aligned} \left\| D_t^\zeta \left( \Pi _{M,N} f\right) -D_{t}^\zeta f\right\| _{L^2(\Omega )} \le \frac{\hat{C_\zeta }N^{(p-r)}}{\Gamma (p-\zeta +1)}\left\| f\right\| _{H^{r}(\Omega )}, \end{aligned}$$

*where*$\hat{C_\zeta }$* is constant. Moreover,*$H^{r}$* is defined according to Definition*3.

### Proof

Using Eq. (5) and the following equation from^43^:

38 $$\begin{aligned} \Vert f*g\Vert _{L^2(\Omega )} \le \Vert f\Vert _1\Vert g\Vert _{L^2(\Omega )}. \end{aligned}$$

Consequently, we derive

39 $$\begin{aligned} \begin{aligned} \left\| D_{t}^\zeta \left( \Pi _{M,N} f\right) -D_{t}^\zeta f\right\| _{L^2(\Omega )}^2&=\left\| I^{p-\zeta }\left( D_{t}^{p}\left( \Pi _{M,N} f(x, t)\right) -D_{t}^{p} f(x, t)\right) \right\| _{L^2(\Omega )}^2, \\&=\parallel \frac{1}{t^{1+\zeta -p}\Gamma (p-\zeta )}*\left( D_{t}^{p}\left( \Pi _{M,N}f(x,t) \right) -D_{t}^{p}f(x,t) \right) \parallel _{L^{2}(\Omega )}^{2},\\&\le \Vert \frac{1}{t^{1+\zeta -p}\Gamma (p-\zeta )} \Vert _{1}^2\left\| D_{t}^{p}\left( \Pi _{M,N} f(x,t)\right) -D_{t}^{p} f(x,t)\right\| _{L^2(\Omega )}^2.\\ \end{aligned} \end{aligned}$$

Based on previous Theorem, we have:

40 $$\begin{aligned} \begin{aligned} \qquad&\le \left( \frac{\hat{C_\zeta }N^{(p-r)}}{\Gamma (p-\zeta +1)}\left\| f\right\| _{H^{r}(\Omega )}\right) ^2. \end{aligned} \end{aligned}$$

Therefore,

$$\begin{aligned} \left\| D_t^\zeta \left( \Pi _{M,N} f\right) -D_{t}^\zeta f\right\| _{L^2(\Omega )} \le \frac{\hat{C_\zeta }N^{(p-r)}}{\Gamma (p-\zeta +1)}\left\| f\right\| _{H^{r}(\Omega )}. \end{aligned}$$

$$\square$$

### Error bound

We are now prepared to determine the error bound of the proposed technique. To accomplish this, let’s rephrase the model described by Eqs. (2) and (3) as follows:

41 $$\begin{aligned} D^\zeta U(t) = g(t,U(t)),t \in [0,\tau ], 0 < \zeta \le 1, \end{aligned}$$

and

42 $$\begin{aligned} U(0)=U_0, \end{aligned}$$

where $U(t) = [H(t),I(t),V(t),L(t)]$ is exact solution and

43 $$\begin{aligned} \begin{aligned}&g(t,U(t))=[g_1 (t,U(t)),g_2 (t,U(t)),g_3 (t,U(t)),g_4 (t,U(t))],\\&U_0=[\nu _1,\nu _2,\nu _3,\nu _4]. \end{aligned} \end{aligned}$$

Suppose $U_N(t)$ represents the approximate solution derived from the proposed method for the model (2)-(3), and *N* is the number of collocation points. Additionally, $e_N (t)$ denotes the error between *U*(*t*) and $U_N(t)$, expressed as $e_N (t) =U(t)-U_N (t)$. By replacing $U_N(t)$ into Eq. (41), we obtain:

44 $$\begin{aligned} Res(t,U_N(t))=D^\zeta U_N (t)- g(t,U_N (t)), \end{aligned}$$

and

45 $$\begin{aligned} U_N(0)=U_0. \end{aligned}$$

We have now reached a stage where we are able to compute the upper bound of the residual function. To continue, we present the following theorem.

#### Theorem 4

*Let* *g(* *t*, *U* *(t)) as defined (*43*) satisfies the Lipschitz condition, and let*$U(t)\in B^r(\Omega )$*. There are constants*$\hat{C_\zeta }$*and C such that:*

46 $$\begin{aligned} \begin{aligned} \Vert Res(t,U_N(t))\Vert _{L^2(\Omega )}&\le \frac{\hat{C_\zeta }N^{(p-r)}}{\Gamma (p-\zeta +1)}\left\| f\right\| _{H^{r}(\Omega )}+\\&C N^{-r}\Vert f\Vert _{H^{r}(\Omega )}. \end{aligned} \end{aligned}$$

where $$n_\zeta <r\le N+1$$, $$n_\zeta -1<\zeta \le n_\zeta =\lceil \zeta \rceil$$ and $$r\in \mathbb {N}$$.

#### Proof

Since *g*(*t*, *U*(*t*)) satisfies the Lipschitz condition, we obtain:

47 $$\begin{aligned} \Vert g(t,U(t))-g(t,U_N(t))\Vert _{L^2(\Omega )} \le B\Vert U(t)-U_N(t)\Vert _{L^2(\Omega )}, \end{aligned}$$

where *B* is constant. If we subtract Eq. (44) from Eq. (41), then we get.

48 $$\begin{aligned} Res(t,U_N(t))=g(t,U(t))- g(t,U_N(t))+D^\zeta e_N (t), \end{aligned}$$

with the initial condition

49 $$\begin{aligned} e_{N}(0)=0. \end{aligned}$$

By applying the norm to both sides of Eq. (48) and using Eq. (47), we obtain:

50 $$\begin{aligned} \begin{aligned} \Vert Res(t,U_N(t))\Vert _{L^2(\Omega )}&=\Vert g(t,U(t))- g(t,U_N (t))+D^\zeta e_N (t) \Vert _{L^2(\Omega )}\\&\le \Vert g(t,U(t))- g(t,U_N (t))\Vert _{L^2(\Omega )}+\Vert D^\zeta e_N (t)\Vert _{L^2(\Omega )}\\&\le B\Vert U(t)-U_N(t)\Vert _{L^2(\Omega )}+|D^\zeta e_N (t)\Vert _{L^2(\Omega )} \end{aligned} \end{aligned}$$

Based on the results of Theorems 1 and 3, there are constants $C_{\zeta }$ and $\lambda$ such that

51 $$\begin{aligned} \begin{aligned} \Vert Res(t,U_N(t))\Vert _{L^2(\Omega )}&\le \frac{\hat{C_\zeta }N^{(p-r)}}{\Gamma (p-\zeta +1)}\left\| f\right\| _{H^{r}(\Omega )}+\\&B\lambda N^{-r}\Vert f\Vert _{H^{r}(\Omega )}. \end{aligned} \end{aligned}$$

where $p<r\le N+1$, $p-1<\zeta \le p=\lceil \zeta \rceil$, and $r\in \mathbb {N}$. Assuming $C=B\lambda$, the theorem is proved. $\square$

Based on the above theorem, we conclude that $\left\| Res(t,U_N(t))\right\| \rightarrow 0$ as $N \rightarrow \infty$. Therefore, $U_{N}(t)$ approaches *U*(*t*).

## Numerical results

Here, we use the proposed method to solve the model (2–3) and set the number of basis functions to 8. All calculations are performed using MATLAB 2022 software on a laptop with 16*GB*, *DDR*4 memory and 12*th* Gen Intel $Core^{TM} i7$ processor. The initial values of the parameters, as explained in Tables 2, 3, 4 and 5, are considered in the interval [0, 1] as follows:

52 $$\begin{aligned} \begin{array}{lll} \mu =6, (L)_{\max }=2200, \tau =1, k=varies, k_1=varies, s=0.0003,\\ \gamma =0.0004, \kappa =0.00004, \eta _T=0.6, \eta _L=0.006, \eta _N=0.0005, \eta _A=0.05, \end{array} \end{aligned}$$

**Fig. 2:**
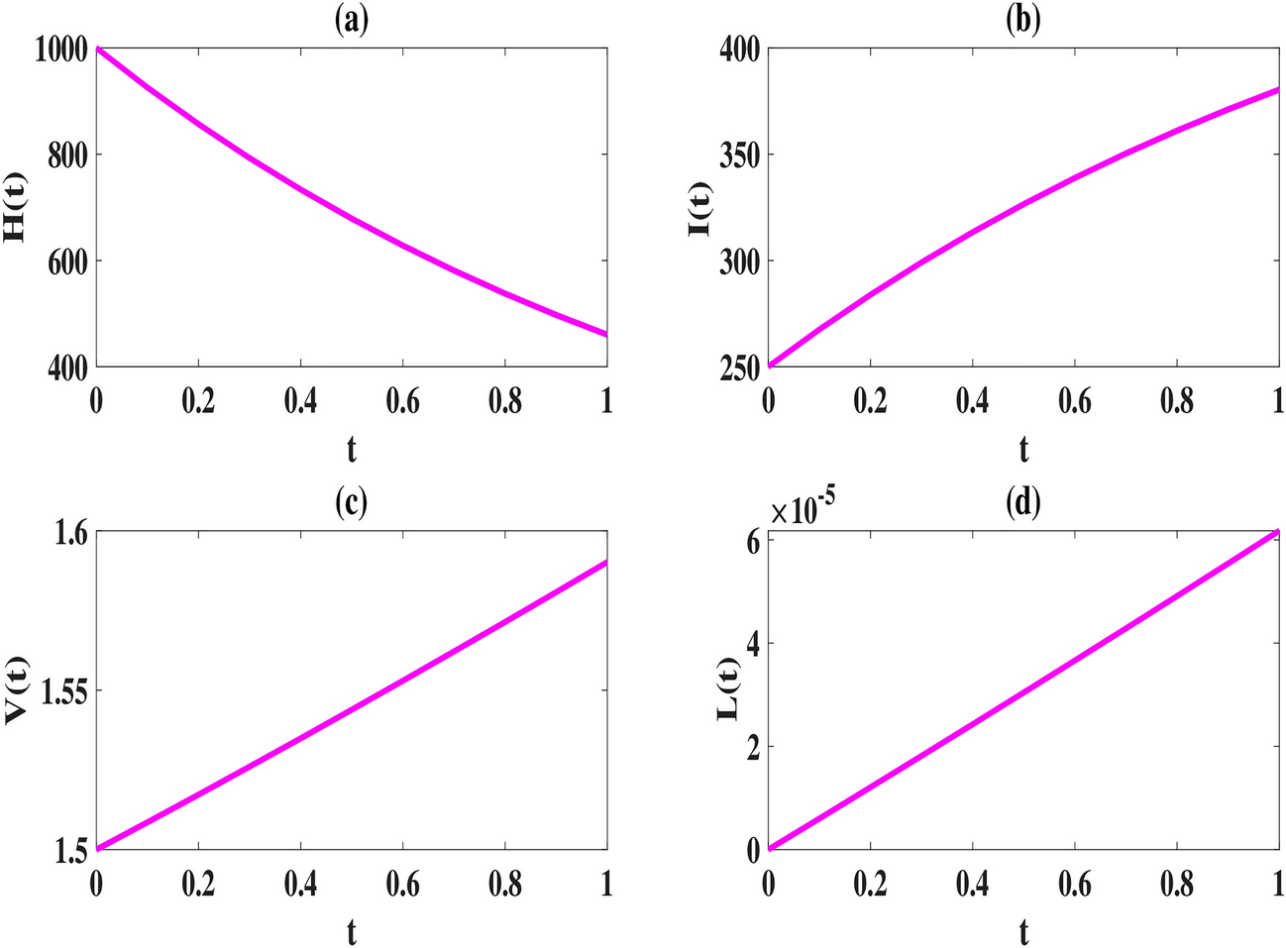
Estimated solutions of *H*(*t*), *I*(*t*), *V*(*t*) and *L*(*t*) for $N=6,\, k=k_1=0.1$ and $\zeta =1$.

**Fig. 3:**
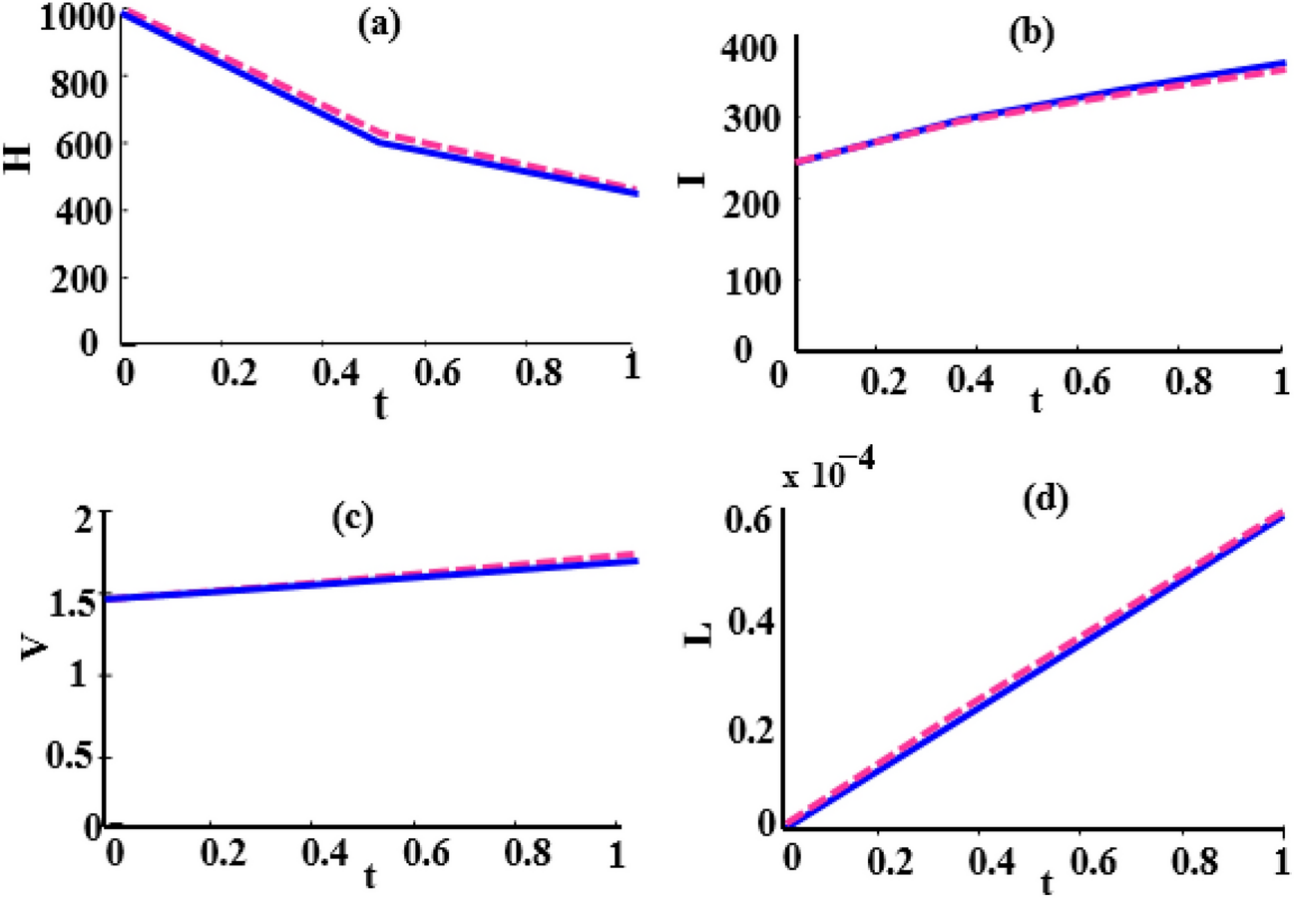
Comparison of solutions obtained by the N-ADM method (solid line) and the fourth-order Runge-Kutta method (dashed line) for *H*(*t*), *I*(*t*), *V*(*t*),  and *L*(*t*) with parameters $k=k_1=0.1$ and $\zeta =1$^34^.

**Fig. 4:**
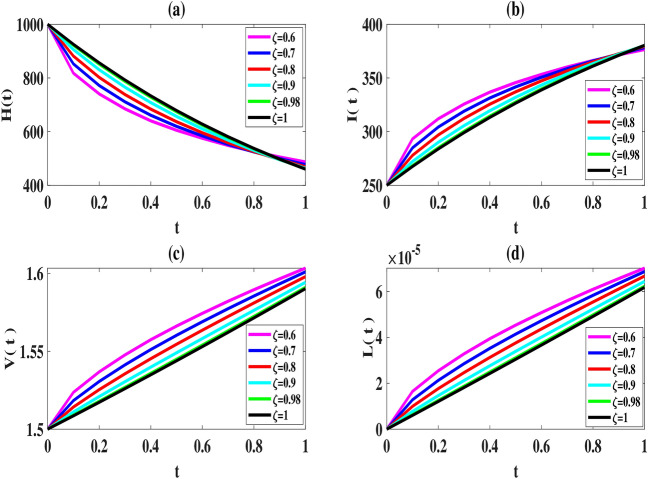
Estimated solutions of model (2) for $N=6,\, k=k_1=0.1, \,$ and various values of $\zeta$.

**Fig. 5:**
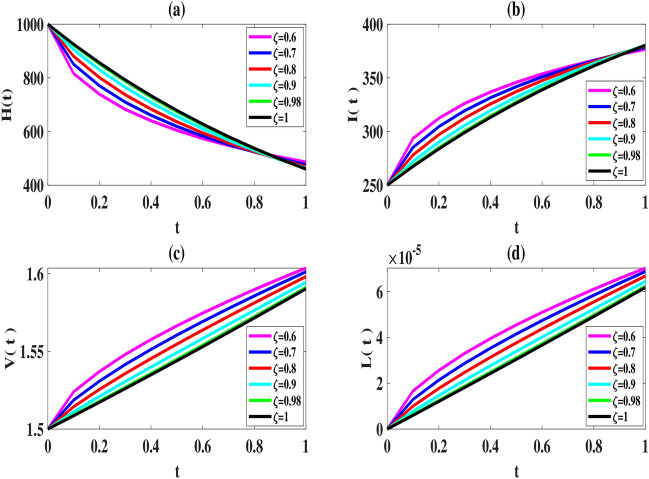
Estimated solutions of model (2) for $N=8,\, k=k_1=0.1$ and various values of $\zeta$.

To compare our results with those obtained using the Natural-Adomian Decomposition method (N-ADM)^34^, we set $\zeta =1$. As anticipated, the graphical results in Fig. 2 align with those presented in^34^ (Fig. 3). Additionally, we approximate *H*(*t*), *I*(*t*), *V*(*t*), and *L*(*t*) using the numerical approach outlined in Sect. “The proposed method” for $N = 6,8$, and different values of $\zeta$. When $\zeta$ approaches one, the solutions of the fractional model system converge to those of the first-order model, as shown in Figs. 4 and 5.

**Fig. 6:**
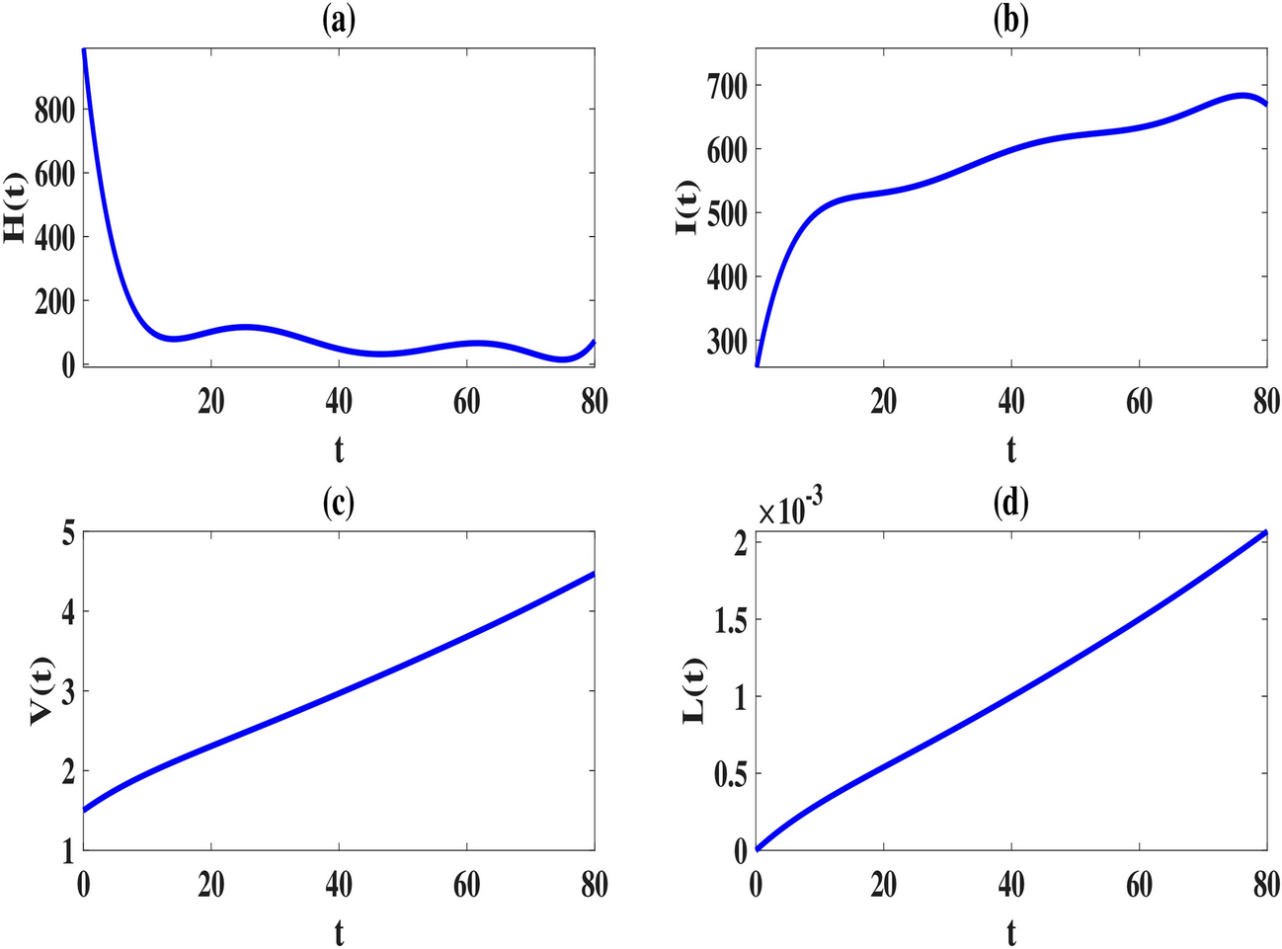
Estimated solutions of model (2) for $N=8,\, k=k_1=0.1$ and $\zeta =1$.

From a biological standpoint, we expect the number of healthy cells to decrease while the populations of latently infected, actively infected, and leukemic cells increase over time. The results of the proposed method in Figs. 4, 5 and 6 are consistent with this theoretical expectation.

Since the model has no exact solution, we employ a comparative approach to assess the error associated with each solution. We compare it with a reference solution obtained through numerical computation using a smaller number of collocation points. The error, denoted as $E_{N,2}$, is determined by calculating the norm of the difference between the solutions obtained with 2*N* and *N* collocation points, represented as $U_{2N}$ and $U_{N}$, respectively. Mathematically, the error can be expressed as:

53 $$\begin{aligned} E_{N,2}=\Vert U_{2N}-U_{N}\Vert _{\infty }. \end{aligned}$$

The rate of convergence is computed by:

54 $$\begin{aligned} R_{N}=log_{2}\left( \frac{E_{N,2}}{E_{2N,2}}\right) . \end{aligned}$$

It is important to note that since we only possess knowledge of the exact “solution” at the mesh points, we employ linear interpolation techniques to estimate solutions at other points. This interpolation enables us to extend our understanding of the solution beyond the mesh points, facilitating a comprehensive evaluation of the error across the entire domain. By employing this methodology, we can effectively quantify the disparities between the computed solutions and the reference solution, providing valuable insights into the accuracy and reliability of our numerical approach.

The error and convergence rate of the proposed method are shown in Tables 2, 3, 4 and 5 for different values of $\zeta$. As expected, increasing the number of collocation points leads to fewer errors while preserving the order of convergence.

**Table 2:** Rate of convergence $R_N$ and Numerical errors $E_{N,2}$ for $\zeta =0.6$.

*N*	H(t)		I(t)		V(t)		L(t)	
	$\Vert .\Vert _{\infty }$	$R_N$	$\Vert .\Vert _{\infty }$	$R_N$	$\Vert .\Vert _{\infty }$	$R_N$	$\Vert .\Vert _{\infty }$	$R_N$
2	$2.120e-1$	1.005	$3.414e-2$	0.941	$2.052e-2$	0.705	$3.712e-3$	1.063
4	$1.055e-1$	0.975	$1.777e-2$	0.836	$1.258e-2$	0.772	$1.777e-3$	0.921
8	$5.370e-2$	0.963	$9.394e-3$	0.859	$7.364e-3$	0.804	$9.378e-4$	1.038
16	$2.754e-2$	1.094	$5.178e-3$	0.889	$4.217e-3$	0.882	$5.164e-4$	1.104
32	$1.290e-2$	0.999	$2.790e-3$	0.888	$2.288e-3$	0.886	$2.401e-4$	1.106
64	$6.453e-3$	1.025	$1.511e-3$	0.884	$1.238e-3$	0.885	$1.115e-4$	1.105
128	$3.171e-3$	1.074	$8.163e-4$	0.885	$6.701e-4$	0.884	$5.181e-5$	1.111
256	$1.506e-3$	−	$4.009e-4$	−	$3.629e-4$	−	$2.398e-5$	−

**Table 3:** Rate of convergence $R_N$ and numerical errors $E_{N,2}$ for $\zeta =0.7$.

*N*	H(t)		I(t)		V(t)		L(t)	
	$\Vert .\Vert _{\infty }$	$R_N$	$\Vert .\Vert _{\infty }$	$R_N$	$\Vert .\Vert _{\infty }$	$R_N$	$\Vert .\Vert _{\infty }$	$R_N$
2	$1.825e-1$	0.953	$1.103e-2$	0.827	$1.845e-1$	0.869	$3.772e-2$	1.101
4	$9.423e-2$	0.961	$6.218e-3$	0.743	$1.010e-1$	0.946	$1.757e-2$	1.020
8	$4.841e-2$	1.038	$3.714e-3$	0.786	$5.241e-2$	0.971	$8.664e-3$	1.109
16	$2.357e-2$	0.995	$2.151e-3$	0.773	$2.674e-2$	0.960	$4.017e-3$	1.102
32	$1.191e-2$	1.005	$1.258e-3$	0.775	$1.374e-2$	0.962	$1.871e-3$	1.107
64	$5.931e-3$	1.055	$7.351e-4$	0.779	$7.051e-3$	0.966	$8.686e-4$	1.103
128	$2.853e-3$	1.041	$4.283e-4$	0.776	$3.608e-3$	0.963	$4.043e-4$	1.108
256	$1.386e-3$	−	$2.501e-4$	−	$1.850e-3$	−	$1.911e-4$	−

**Table 4:** Rate of convergence $R_N$ and numerical errors $E_{N,2}$ for $\zeta =0.8$.

*N*	H(t)		I(t)		V(t)		L(t)	
	$\Vert .\Vert _{\infty }$	$R_N$	$\Vert .\Vert _{\infty }$	$R_N$	$\Vert .\Vert _{\infty }$	$R_N$	$\Vert .\Vert _{\infty }$	$R_N$
2	$1.827e-1$	0.867	$2.664e-3$	0.945	$2.246e-2$	0.869	$2.954e-3$	1.024
4	$1.011e-1$	0.977	$1.383e-3$	0.930	$1.264e-2$	0.959	$1.452e-3$	1.010
8	$5.137e-2$	0.992	$7.358e-4$	0.952	$6.501e-3$	0.985	$7.213e-4$	0.984
16	$2.582e-2$	0.972	$3.801e-4$	0.947	$3.283e-3$	0.979	$3.724e-4$	0.988
32	$1.315e-2$	0.979	$1.871e-4$	0.951	$1.665e-3$	0.980	$1.877e-4$	1.059
64	$6.671e-3$	0.974	$9.973e-5$	0.949	$8.440e-4$	0.982	$8.670e-5$	1.027
128	$3.395e-3$	0.974	$5.010e-5$	0.950	$4.272e-4$	0.981	$4.254e-5$	1.008
256	$1.728e-3$	−	$2.593e-5$	−	$2.163e-4$	−	$1.115e-5$	−

**Table 5:** Rate of convergence $R_N$ and numerical errors $E_{N,2}$ for $\zeta =0.9$.

*N*	H(t)		I(t)		V(t)		L(t)	
	$\Vert .\Vert _{\infty }$	$R_N$	$\Vert .\Vert _{\infty }$	$R_N$	$\Vert .\Vert _{\infty }$	$R_N$	$\Vert .\Vert _{\infty }$	$R_N$
2	$2.003e-1$	1.146	$1.827e-2$	0.729	$1.614e-1$	0.959	$1.853e-2$	0.983
4	$1.357e-1$	1.113	$1.102e-2$	0.897	$8.078e-2$	0.888	$9.378e-3$	1.046
8	$6.274e-2$	1.088	$5.915e-3$	0.987	$2.784e-2$	0.867	$4.542e-3$	1.007
16	$4.291e-2$	1.022	$2.984e-3$	0.981	$1.526e-2$	0.857	$2.258e-3$	1.126
32	$2.113e-2$	1.063	$1.511e-3$	0.980	$8.421e-3$	0.868	$1.034e-3$	1.132
64	$1.010e-2$	1.097	$7.657e-4$	0.987	$4.613e-3$	0.863	$4.718e-4$	1.120
128	$4.719e-3$	1.076	$3.851e-4$	0.989	$2.535e-3$	0.862	$2.185e-4$	1.116
256	$2.238e-3$	−	$1.945e-4$	−	$1.394e-3$	−	$1.011e-4$	−

## Conclusion

In this study, we investigated Caputo’s type of fractional HTLV-I model. We proposed a numerical method that combines the operational matrix and the spectral method to solve this system effectively. We established the effectiveness and accuracy of the proposed method through theoretical error analysis and numerical results. Comparisons with existing methods further demonstrated the superior performance and reliability of our approach. A key advantage of our method is its fast convergence rate and low computational cost, which are attributable to the inherent sparsity of the derivative operational matrices associated with Legendre polynomials. Additionally, our analysis of graphical results revealed an important finding: the order of the time-fractional derivative ($\zeta$) significantly influences the numerical approximation. As $\zeta$ approaches 1, the results obtained using the fractional model converge to those of the standard model (at $\zeta =1$). This observation highlights the model’s ability to capture the transition between fractional and integer-order dynamics. Future research could explore the effectiveness of combining operational matrices with other spectral methods, such as Galerkin and Tau, to solve the fractional model and compare these approaches with the proposed method. Additionally, investigating the efficiency of this method for solving space-time differential equation systems would be another valuable direction for future study.

## Data Availability

The data sets used and/or analyzed during the current study available from the corresponding author on reasonable request.

## References

[1] Sajjadi, S., Hejazi, S., Ravanshad, S. & Jafarzadeh, R. Human T-lymphotropic virus type 1 and novel coronavirus disease 2019, More complex than just a simple coinfection. *Gene* **834**, 146550 (2022). 35569772 10.1016/j.gene.2022.146550 PMC9098513

[2] Ramanayake, S., Moulding, D. A., Tanaka, Y. A. & Singh & Bangham, C. R. M.,. *Dynamics and consequences of the HTLV-1 proviral plus-strand burst* 18 (PLoS, Pathog, 2022). 10.1371/journal.ppat.1010774 PMC9731428 36441826

[3] Farmanbar, A., Firouzi,S. & Park, S. J. et al, Multidisciplinary insight into clonal expansion of HTLV-1 infected cells in adult T-cell leukemia via modeling by deterministic finite automata coupled with high-throughput sequencing.*BMC. Med. Genomics.* **10**, (2017). 10.1186/s12920-016-0241-2 PMC5282739 28137248

[4] Kalinichenko, S., Komkov, D. & Mazurov, D. HIV-1 and HTLV-1 transmission modes: Mechanisms and importance for virus spread. *Viruses.* **14**, (2022). 10.3390/v14010152 PMC8779814 35062355

[5] Song, C. & Xu, R. Mathematical analysis of an HTLV-I infection model with the mitosis of T-cells and delayed CTL immune response. *Nonlinear Anal. Model.* **26**, 1–20 (2021).

[6] Mohanty, S. & Harhaj, E. W. Mechanisms of Innate Immune Sensing of HTLV-1 and Viral Immune Evasion. *Pathog.* **12**, 735 (2023). 10.3390/pathogens12050735 PMC10221045 37242405

[7] AlShamrani, N. H. Stability of an HTLV-HIV coinfection model with multiple delays and CTL-mediated immunity Adv. *Differ. Equ.* **2021**, 270 (2021). 10.1186/s13662-021-03416-7 PMC8144699 34054935

[8] Perelson, A. S. & Nelson, P. W. Mathematical analysis of HIV-1 dynamics in vivo. *SIAM REV.* **41**(1), 3–44 (1999).

[9] Bangham, C. R. M. The immune control and cell-to-cell spread of human T-lymphotropic virus type 1. *J. Gen. Virol.* **84**, 3177–3189 (2003). 14645900 10.1099/vir.0.19334-0

[10] Poiesz, B. J. et al. Detection and isolation of type C retrovirus particles from fresh and cultured lymphocytes of a patient with cutaneous T-cell lymphoma. *Proc. Natl. Acad. Sci USA* **77**(12), 7415–7419 (1980). 6261256 10.1073/pnas.77.12.7415 PMC350514

[11] Matsuoka, M. & Jeang, K. T. Human T-cell leukaemia virus type 1 (HTLV-I) infectivity and cellular transformation. *Nat. Rev. Cancer* **7**, 270–280 (2007). 17384582 10.1038/nrc2111

[12] Wang, W. & Ma, W. Global dynamics of a reaction and diffusion model for an HTLV-I infection with mitotic division of actively infected cells. *J. Appl. Anal. Comput.* **7**, 899–930 (2017).

[13] Chen, S., Liu, Z., Wang, L. & Zhang, X. Global dynamics analysis for a nonlinear HTLV-I model with logistic proliferation and CTL response. *Int. J. Biomath.* **17**(03), 2350023 (2024).

[14] Cook, L. B., Elemans, M., Rowan, A. G. & Asquith, B. HTLV-1: Persistence and pathogenesis. *Virol.* **435**, 131–140 (2013). 10.1016/j.virol.2012.09.028 23217623

[15] Vieira, B. A. et al. Prevalence of human T-lymphotropic virus type 1 and 2 (HTLV-1/-2) infection in pregnant women in Brazil: A systematic review and meta-analysis. *Sci. Rep.* **11**, 15367 (2021). 34321555 10.1038/s41598-021-94934-7 PMC8319321

[16] Kamoi, K. et al. Horizontal transmission of HTLV-1 causing uveitisI. *Lancet Infect. Dis.* **21**, 578 (2021). 33773136 10.1016/S1473-3099(21)00063-3

[17] Khajanchi, S., Bera, S. & Kumar Roy, T. Mathematical analysis of the global dynamics of a HTLV-I infection model, considering the role of cytotoxic T-lymphocytesT. *Math. Comput. Simul.* **180**, 354–378 (2021).

[18] Li, V. & Shu, H. Multiple stable periodic oscillations in a mathematical model of CTL response to HTLV-I infection. *Bull. Math. Biol.* **73**, 1774–1793 (2011). 20976566 10.1007/s11538-010-9591-7

[19] Li, F. & Ma, W. Dynamics analysis of an HTLV-1 infection model with mitotic division of actively infected cells and delayed CTL immune response. *Math. Methods Appl. Sci.* **41**, 3000–3017 (2018).

[20] Elaiw, A. M. & AlShamrani, N. H. Analysis of a within-host HTLV-I/HIV-1 co-infection model with immunity. *Virus Res.* **295**, 1–23 (2021). 10.1016/j.virusres.2020.198204 33157165

[21] Stilianakis, N. I. & Seydel, J. Modeling the T-cell dynamics and pathogenesis of HTLV-I infection. *B. Math. Biol.* **61**, 935–947 (1999). 10.1006/bulm.1999.0117 17886750

[22] Katri, P. & Shigui, R. Dynamics of human T-cell lymphotropic virus I (HTLV-I) infection of T-cell. *C. R. Biol.* **327**, 1009–1016 (2004). 15628223 10.1016/j.crvi.2004.05.011

[23] Arfan, M. et al. A novel semi-analytical method for solutions of two dimensional fuzzy fractional wave equation using natural transform. *Dyn. Syst. Ser.* **15**, 315–338 (2022).

[24] Nikan, O., Avazzadeh, Z. & Tenreiro Machado, J. A. An improved localized radial basis-pseudospectral method for solving fractional reaction-subdiffusion problem. *Results Phys.* **23**, 104048 (2021).

[25] Sayevand, K. On a flexible extended homotopy perturbation method and its applications in applied chemistry. *J. Math. Chem.* **58**, 1291–1305 (2020).

[26] Amin, R., Shah, K., Asif, M. & Khan, I. Efficient numerical technique for solution of delay Volterra-Fredholm integral equations using Haar wavelet. *Heliyon* **6**, e05108 (2020). 33083601 10.1016/j.heliyon.2020.e05108 PMC7553982

[27] Sharma, S., Kumar, S., Pandey, R. K. & Kumar, K. Two-dimensional collocation method for generalized partial integro-differential equations of fractional order with applications. *Math. Method. Appl. Sci.* **46**, 12155–12175 (2023).

[28] Kumar, S., Pandey, R. K., Kumar, K., Kamal, S. & Dinh, T. N. Finite difference collocation method for the generalized fractional diffusion equation. *Fract. Fract.* **6**(7), 387 (2022).

[29] Mehri, A., Bouhadjera, H., Abdo, M. S., Alzumi, H. Z. & Shammakh, W. Finite element method for fractional order parabolic obstacle problem with nonlinear source term. *Partial Differ. Equ. Appl. Math.* **10**, 2666–8181 (2024).

[30] Kumar, S., Kumar, K., Pandey, R. K. & Xu, Y. Legendre collocation method for new generalized fractional advection-diffusion equation. *Int. J. Math.* **101**, 1–23 (2024).

[31] Ghoreishi, F., Ghaffari, R. & Saad, N. Fractional Order Rung-Kutta Methods. *Fract. Fract.* **7**, 245 (2023).

[32] Ahmed, S., Jahan, S., Shah, K. & Abdeljawad, T. On mathematical modelling of measles disease via collocation approach. *AIMS Publ. Health.Bold">11*, 628–653 (2024). 10.3934/publichealth.2024032 PMC11252575 39027389

[33] Rashidinia, J., Momeni, A. & Molavi-Arabshahi, M. Solution of convection-diffusion model in groundwater pollution. *Sci. Rep.* **14**(1), 2075 (2024). 38267523 10.1038/s41598-024-52393-w PMC10808552

[34] Rida, S. Z. & Gaber, Y. A. Stability analysis of generalized model of human T-cell lymphotropic virus I (HTLV-I) infection of -cells -cells. *J. Fract. Calc. Appl Anal* **11**, 170–181 (2020).

[35] Erturk, V. S., Odibat, Z. M. & Momani, S. An approximate solution of a fractional order differential equation model of human T-cell lymphotropic virus I (HTLV-I) infection of -cells. *Comput. Math. Appl.* **62**, 996–1002 (2011).

[36] Arafa, A. A. M., Rida, S. Z. & Khalil, M. Fractional order model of human T-cell lymphotropic virus I (HTLV-I) infection of -cells. *Adv. Stud. Biol.* **3**, 347–353 (2011).

[37] Rashidinia, J., Molavi-Arabshahi, V. & Yousefi, M. An efficient approach for solving a class of fractional anomalous diffusion equation with convergence. *Phys. Scr.Bold">99*, 628–653 (2024).

[38] Yang, Y., Ma, Y. & Wang, L. Legendre polynomials operational matrix method for solving fractional partial differential equations with variable coefficients. *Math. Probl. Eng.* **2015**, 915195 (2015).

[39] Pourbabaee, M. & Abbas Saadatmandi, A. A novel Legendre operational matrix for distributed order fractional differential equations. *Appl. Math. Comput.* **361**, 215–231 (2019).

[40] Yang, X. J. *General fractional derivatives: Theory methods and applications* (CRC Press, 2019).

[41] Shen, J., Tang, T. & Wang, L. *Algorithms, analysis and applications* (Springer, 2010).

[42] Canuto, C., Hussaini, M. Y., Quarteroni, A. & Zang, T. A. *Spectral methods: Fundamentals in single domains* (Springer-Verlag, 2006).

[43] Mashayekhi, S. & Razzaghi, M. Numerical solution of distributed order fractional differential equations by hybrid functions. *J. Comput. Phys.* **315**, 169–181 (2016).

